# Sunscreen Use on the Dorsal Hands at the Beach

**DOI:** 10.1155/2013/269583

**Published:** 2013-06-06

**Authors:** Donald B. Warren, Ryan R. Riahi, Jason B. Hobbs, Richard F. Wagner

**Affiliations:** ^1^Department of Dermatology, The University of Texas Medical Branch, 301 University Boulevard, Galveston, TX 77555-0783, USA; ^2^Department of Dermatology, Louisiana State University, New Orleans, LA 70112-2865, USA

## Abstract

*Background.* Since skin of the dorsal hands is a known site for the development of cutaneous squamous cell carcinoma, an epidemiologic investigation was needed to determine if beachgoers apply sunscreen to the dorsal aspect of their hands as frequently as they apply it to other skin sites. *Aim.* The aim of the current study was to compare the use of sunscreen on the dorsal hands to other areas of the body during subtropical late spring and summer sunlight exposure at the beach. *Materials and Methods.* A cross-sectional survey from a convenience sample of beachgoers was designed to evaluate respondent understanding and protective measures concerning skin cancer on the dorsal hands in an environment with high natural UVR exposure. *Results.* A total of 214 surveys were completed and analyzed. Less than half of subjects (105, 49%) applied sunscreen to their dorsal hands. Women applied sunscreen to the dorsal hands more than men (55% women versus 40% men, *P* = 0.04). Higher Fitzpatrick Skin Type respondents were less likely to protect their dorsal hands from ultraviolet radiation (*P* = 0.001). *Conclusions.* More public education focused on dorsal hand protection from ultraviolet radiation damage is necessary to reduce the risk for squamous cell carcinomas of the hands.

## 1. Introduction

Sunlight exposure, particularly ultraviolet radiation (UVR), is recognized as a risk factor for skin cancer. Photoprotection may reduce the risk of developing skin cancer. However, what efforts are made to protect the dorsal aspect of hands from UVR is unknown.

There are over a million cases of skin cancer diagnosed each year in the United States (US), and this number is expected to double in the next 30 years [1–3]. About 2000 deaths occur each year from nonmelanotic skin cancers in the US. The cost of care for nonmelanoma skin cancers is the fifth highest for all cancers in the US Medicare population [4]. Many factors contribute to the risk for developing skin cancer including UV exposure, low Fitzpatrick Skin Types, male gender, and advanced age. However, unprotected UVR exposure is the single most important environmental risk factor for developing most nonmelanocytic skin cancers.

Several studies have evaluated photoprotective behaviors on anatomic regions that are frequently partially or completely unprotected from UVR damage, such as the scalp, lips, and eyelids [5–7]. The dorsal hands, along with the face, forearms, neck, and legs receive the most sunlight exposure, but unlike the forearms and legs, the hands are often unprotected by clothing. In lower latitudes during the midday hours, the dorsal hands are exposed to more sunlight than any other body part [8]. This additional UVR exposure may put skin of the dorsal hand at increased risk for developing skin cancer. Squamous cell carcinoma (SCC) is more common than basal cell carcinoma on the dorsal hand. It has been estimated that 58–90% of all hand malignancies are due to SCC [9]. Some investigators cite metastasis rates of 2–5% for SCC, while others estimate 10–28% [10, 11]. Local recurrence rates for SCC on the dorsal hands are as high as 22–28% [11, 12]. Men have a much higher incidence of cancer on the dorsal hands than women. One study found that the ratio of male to female SCC is 3 : 1 in this location [13]. Contributing factors to the current male predominance of dorsal hand SCC are the historic male predominance in many outdoor professions and some outdoor recreational activities such as fishing and golf. Men might also be less likely to wear sunscreen on their hands or use lotions with a sun protection factor (SPF) rating.

The primary goal of this survey was to determine if beachgoers protected the dorsal skin of their hands as frequently as other areas of their skin. The secondary goal of this study was to collect additional information about subject demographics and knowledge about skin cancer to determine if these variables were significantly correlated with UVR dorsal hand protection.

## 2. Materials and Methods

Following approval of the study protocol by The University of Texas Institutional Review Board, three of the authors (DW, RR, and JH) distributed anonymous questionnaires developed by authors (the appendices) to a convenience sample of beachgoers on Galveston Island, TX, public beaches who were at least 18 years of age. Data were gathered from late May to early September during 2011 on days with an average temperature greater than 80 F and with less than 50% cloud coverage. Data were collected about subjects' age, gender, Fitzpatrick Skin Type (FST), UVR skin and hand protection behaviors, duration of time spent at the beach when the survey was performed, and other protective behaviors including hats, umbrellas, shirts, and lip protection, and baseline knowledge questions regarding skin and hand cancer. In order to assess dorsal hand protection among different age ranges, participants were grouped into one of the 4 categories: 18–34 years old, 35–49 years old, 50–64 years old, and 65 years old or older. Associations between two factors were assessed using the Pearson chi-square test. The chi-square test was assessed at the 0.05 level of significance. Data analyses were carried out using PROC FREQ in the SAS system, release 9.2 [14].

## 3. Results

A total of 214 questionnaires were completed and analyzed. The average age of respondents was 40 years old and ranged from 18 to 86 years. The self-reported FSTs among the study population were 4% Type 1, 19% Type 2, 41% Type 3, 22% Type 4, 9% Type 5, and 5% Type 6. The questionnaires included data from 149 participants (70%) who reported sunscreen use somewhere on their bodies and 65 (30%) who did not apply sunscreen anywhere. Among those who used a sunscreen while being at the beach, 65% applied a lotion-based sunscreen and 35% applied a spray. However, the rate of photoprotection to the dorsal hand was not significantly different when comparing lotions versus sprays (*P* = 0.81). Reapplication of sunscreen to the hand was also not affected by the type of sunscreen used (*P* = 0.52). Fewer than 65% of all responders applied sunscreen to their forearms while 35% did not.

Regarding the dorsal hands, 105 (49%) applied sunscreen while 109 (51%) did not. There was a significant difference (*P* = 0.009) between men and women with rates of sunscreen use in general (60% men versus 77% women) and sunscreen use to the dorsal hands (40% men versus 55% women, Table 1, *P* = 0.04).

**Figure 1:**
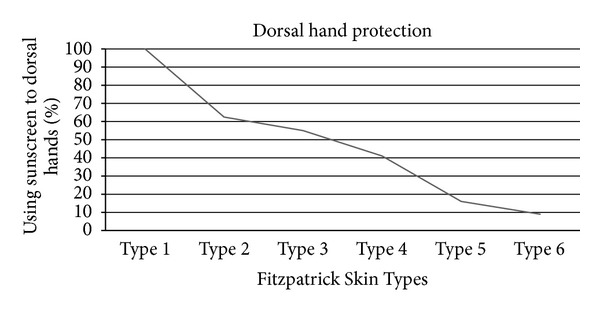
UVR dorsal hand protection in relation to Fitzpatrick Skin Types.

Only 21% of the respondents reapplied sunscreen during their stay at the beach, but less (13%) reapplied it to the dorsal hands. Length of stay at the beach affected rates of overall sunscreen use: 57% of those who stayed less than 2 hours applied sunscreen compared to 89% of those who stayed longer than 4 hours (*P* = 0.0002) but was not significantly different for dorsal hand protection. Those who stayed less than 2 hours exhibited a 45% dorsal hand protection rate, while 49% of those staying longer than 4 hours protected their hands (*P* = 0.46). When the forearm, an adjacent area, had received photoprotection, 21% of that group did not protect the dorsal hands. The most common reason given for not protecting the back of the hands was “did not think about it,” followed by “did not like the feel of it.” Higher FST respondents were less likely to protect their dorsal hands from UVR (*P* = 0.001). The rates of dorsal hand protection among each FST were 100% of Type 1 respondents who used photoprotection, 62.5% among Type 2 respondents, 55% among Type 3 respondents, 41% among Type 4 respondents, 16% among Type 5 respondents, and 9% among Type 6 respondents (Figure 1).

Photoprotection rates for the dorsal hands among self-identified ethnicities were the highest in Whites (57%), followed by Hispanics (44%), Asians (43%), other (27%), and the lowest in Blacks (7%, *P* = 0.007). Comparing age groups, the 18–34-year-old group was the least likely to protect the dorsal hands (37%, *P* = 0.005). Rates of other photoprotective measures such as cap/hat (45%), umbrella/shade (44%), lip protection (28%), and eyewear (71%) were also analyzed. Of these additional modes of photoprotective behavior, only umbrella/shade use correlated with dorsal hand protection (*P* = 0.001). Rates for hand protection associated with behaviors such as tobacco use and alcohol use were also surveyed. The data demonstrated that subjects engaging in these behaviors were less likely to protect their dorsal hands from UVR. Only 35% of tobacco users applied sunscreen to the backs of their hands compared to 54% of people who did not use tobacco products (*P* = 0.01). Respondents who drank more than 5 alcoholic beverages a week and applied dorsal hand protection (32%) were less likely to apply sunscreen to the hands than those who drank less (56%, *P* = 0.001). Other factors such as sunburns during the year (*P* = 0.21), outdoor occupations (*P* = 0.18), tanning salon use (*P* = 0.89), and personal or family history of skin cancer (*P* = 0.32) did not significantly influence rates of UVR dorsal hand protection.

Four baseline knowledge questions were also conducted during the survey. Question 1 asked if women were more likely to have skin cancer than men (correct answer is no), and 42% responded yes, 36% chose no, and 22% did not know. Question 2 asked if excessive sun exposure is linked to increased incidence of skin cancer (correct answer is yes) with 91% responding yes, 4% responding no, and 5% not knowing. Question 3 inquired if hand cancer is more common in younger people than older people (correct answer is no), and 51% said no, 16% said yes, and 33% did not know. Question 4 asked if hand cancer is more common in men than women (correct answer is yes), and 16% responded no, 46% said yes, and 38% did not know. Correct knowledge of any of the 4 baseline questions was not associated with significantly higher rates of UVR dorsal hand protection (Q1 *P* = 0.52, Q2 *P* = 0.78, Q3 *P* = 0.07, Q4 *P* = 0.62).

## 4. Discussion

Hand cancer is an important skin disease, and its potential outcomes on morbidity and mortality can be substantial [15]. Treatment often requires surgery [16]. Skin cancers on the hand are more likely to metastasize when compared to other nonmelanoma skin cancers affecting different skin areas. Cancers in this location may also be more likely to recur following treatment. UVR has been shown to be a risk factor in the development of nonmelanoma skin cancers involving the dorsal hands. This location typically receives higher amounts of UVR than most other body surface areas and depending on the time of day and latitude and may receive the most amount of UVR compared to all other skin areas. As with other skin areas like the scalp and lips, this study demonstrates that the dorsal hands are a neglected body area for photoprotection when compared to other areas where sunscreen is applied more frequently.

Our study population showed a low rate of dorsal hand photoprotection (49%) in an environment of high UVR exposure, the beach. Statistically significant differences were observed based on FST, ethnicity, gender, and age. Participants with low FSTs were more likely to apply sunscreen and on the dorsal hands compared to higher FST participants. With regards to ethnicity, the highest rates of dorsal hand protection were found in Whites and the lowest in Blacks. Women were more likely to use sunscreen in general and on the dorsal hands compared to their male counterparts. This later finding is important because hand cancer historically affects males more than females by a 3 : 1 ratio. Several factors are likely responsible for this skewed incidence, including traditional workforce gender imbalances. Inadequate photoprotective behaviors by men also may play a role in their higher rates of dorsal hand cancer compared to women.

Total skin protection from UVR exposure is the best primary prevention for decreasing the incidence of UVR related skin cancers, but previous research indicates that specific anatomic locations such as the lips, eyelids, and scalp are less protected by beachgoers. More public education focused on dorsal hand protection from UVR damage is necessary to reduce skin cancers in this location. This educational need is also evident based on responses to the baseline knowledge survey regarding hand cancer. Although the majority of people understand that UVR is a risk factor for skin cancer (91%), only 51% were aware that hand cancer is more common in the elderly and that men are more affected than women (46%). Protection of the dorsal hands from sunlight may also delay or prevent the appearance of photoaging in this anatomic location. Helpful strategies to inform the public about hand cancer could come from pamphlets about skin cancer found in healthcare providers' offices and other health promotion activities. Sunscreen manufacturers could also list commonly neglected areas of photoprotection with the directions for product use. Media campaigns should focus on reaching younger demographics as this study found that the 18–34-year-old population was the least likely to protect the dorsal hands (37%). This younger population would potentially benefit the most from such photoprotective intervention due to less lifetime cumulative exposure to this frequently encountered environmental carcinogen. These preventative efforts may improve public awareness and hopefully lower the future incidence of hand cancers.

## 5. Conclusions

Surveyed Galveston beachgoers were less likely to protect their dorsal hands from UVR injury than other areas of skin. UVR protection of the dorsal hands deserves emphasis in public health messages about skin cancer protection, along with other identified body locations such as the scalp, lips, and eyelids.

## Figures and Tables

**Table 1:** Gender differences in dorsal hand UVR protection (women are more likely to apply sunscreen to their dorsal hands than men, *P* = 0.04).

Gender	Sunscreen %	No sunscreen %
Women	55.37	44.63
Men	40.23	59.77

Total	49.04	50.96

## Appendices

**Table:**

Age (must be 18 or older for survey)

**Table:**

Gender:

**Table:**

(1) Female
(2) Male

**Table:**

Skin Type:

**Table:**

(1) Pale white; always burns, does not tan
(2) White; burns easily, tans with difficulty
(3) Cream/Light Brown; tans after initial sunburn
(4) Brown; burns minimally, tans easily
(5) Dark Brown; rarely burns, tans easily
(6) Black; never burns, tans easily

**Table:**

Ethnicity

**Table:**

(1) Asian/Pacific Islander
(2) Black
(3) Hispanic
(4) White
(5) Other

### A. Sun Protection

**Table:**

Do you currently have sunburn anywhere?

**Table:**

(1) No
(2) Yes

**Table:**

How many hours have you been at the beach today?

**Table:**

(1) 0–2 hours
(2) More than 2 hours but less than 4 hours
(3) More than 4 hours but less than 6 hours
(4) More than 6 hours

**Table:**

Did you apply sunscreen on today? What is SPF?

**Table:**

(1) No
(2) <15 SPF
(3) 15–30 SPF
(4) >30 SPF
(5) Yes, but I don't know what is SPF

**Table:**

If yes, what type of sunscreen did you use?

**Table:**

(1) Lotion
(2) Spray

**Table:**

Did you apply sunscreen to the *back of your hands*?

**Table:**

(1) No
(2) Yes
(3) Not sure

**Table:**

If No, why not?

**Table:**

(1) Did not think about it
(2) Did not like feel of it (oily, greasy, etc.)
(3) Other

**Table:**

Did you apply sunscreen to your *forearms*?

**Table:**

(1) No
(2) Yes
(3) Not sure

**Table:**

If No, why not?

**Table:**

(1) Did not think about it
(2) Did not like feel of it (oily, greasy, etc.)
(3) Other

**Table:**

How many applications of sunscreen did you use on your skin today?

**Table:**

1	2	3	4 or more

**Table:**

How many applications of sunscreen did you use on the *back of your hands* today?

**Table:**

1	2	3	4 or more

**Table:**

How many applications of sunscreen did you use on your *forearms* today?

**Table:**

1	2	3	4 or more

**Table:**

Why did you *re*apply sunscreen to your skin today?

**Table:**

(1) Did not reapply
(2) Time for another application
(3) Sweating/swimming
(4) Skin redness
(5) Other reason? Please tell us

**Table:**

Why did you *re*apply sunscreen to the *back of your hands* today?

**Table:**

(1) Did not reapply
(2) Time for another application
(3) Sweating/swimming
(4) Skin redness
(5) Other reason? Please tell us

**Table:**

Why did you reapply sunscreen to your *forearms* today?

**Table:**

(1) Did not reapply
(2) Time for another application
(3) Sweating/swimming
(4) Skin redness
(5) Other reason? Please tell us

**Table:**

Did you use any of the following forms of skin sun protection today:

**Table:**

Clothing:

**Table:**

(1) only bathing suit
(2) short-sleeved shirt or shorts
(3) long-sleeved shirt or pants

**Table:**

Hat:

**Table:**

(1) none
(2) Cap (no ear protection from sun)
(3) Hat with brim of 3 inches or less
(4) Hat with brim of more than 3 inches

**Table:**

Umbrella/Shade:

**Table:**

(1) No
(2) Yes

**Table:**

Lipstick or Lip Balm with SPF:

**Table:**

(1) No
(2) Yes

**Table:**

Eyewear:

**Table:**

(1) No
(2) Yes

### B. Skin Cancer Risk Factors

**Table:**

How many sunburns have you had this summer?

**Table:**

Sunburn = redness > 24 hours and/or peeling, pain, swelling, blistering

**Table:**

Do you use tobacco products?

**Table:**

(1) No
(2) Yes

**Table:**

Do you have more than 5 alcoholic drinks per week?

**Table:**

(1) No
(2) Yes

**Table:**

While at work, do you spend part of your time outdoors?

**Table:**

(1) No
(2) Yes

**Table:**

Did you use indoor tanning salons in the past year?

**Table:**

(1) No
(2) Monthly
(3) Weekly
(4) Daily

**Table:**

Did you or a family member ever have a skin cancer?

**Table:**

(1) No
(2) Self
(3) Immediate Family
(4) Extended Family
(5) I don't know

**Table:**

Did you or a family member ever have a skin cancer on the *back of the hand*?

**Table:**

(1) No
(2) Self
(3) Immediate Family
(4) Extended Family
(5) I don't know

**Table:**

Did you or a family member ever have a skin cancer on the *forearm*?

**Table:**

(1) No
(2) Self
(3) Immediate Family
(4) Extended Family
(5) I don't know

### C. Baseline Knowledge

**Table:**

(1) Women are more likely to get skin cancer than men?

**Table:**

(1) No
(2) Yes
(3) I don't know

**Table:**

(2) Excessive sun exposure to the skin can increase the risk of skin cancer?

**Table:**

(1) No
(2) Yes
(3) I don't know

**Table:**

(3) Hand cancer is more common in younger people than older people?

**Table:**

(1) No
(2) Yes
(3) I don't know

**Table:**

(4) Men get more hand cancer than women?

**Table:**

(1) No
(2) Yes
(3) I don't know
